# Impact of Race and Geographic Area of Residence on Outcomes After Allogeneic Stem Cell Transplant

**DOI:** 10.3389/fonc.2022.801879

**Published:** 2022-02-25

**Authors:** Audrey M. Sigmund, Qiuhong Zhao, Justin Jiang, Patrick Elder, Don M. Benson, Ashley Rosko, Naresh Bumma, Abdullah Khan, Srinivas Devarakonda, Sumithira Vasu, Samantha Jaglowski, Alice Mims, Hannah Choe, Karilyn Larkin, Jonathan Brammer, Sarah Wall, Nicole Grieselhuber, Ayman Saad, Sam Penza, Yvonne A. Efebera, Nidhi Sharma

**Affiliations:** ^1^Division of Hematology, Department of Internal Medicine, The Ohio State University, Columbus, OH, United States; ^2^College of Medicine, The Ohio State University, Columbus, OH, United States

**Keywords:** race, geographic location of residence, allogeneic transplant, health disparities, GvHD

## Abstract

**Background:**

Allogeneic hematopoietic stem cell transplant (allo-HCT) is a potential curative therapy for a variety of hematologic disorders. However, it requires highly specialized care that is only available at select centers across the country. Thus, minority populations are at risk for healthcare disparities in access to and outcomes of allo-HCT. Our study aimed to assess the impact of race and location of residence on outcomes of allo-HCT.

**Methods:**

We performed a retrospective analysis of all patients who underwent allo-HCT at the Ohio State University from 1984 to 2018. Patients were divided by race (Caucasian, African American, and other) and grouped by zip code into rural, suburban, and urban groups. Primary endpoints included progression-free survival (PFS) and overall survival (OS).

**Results:**

Of the 1,943 patients included in the study, 94.3% self-identified as Caucasian, 4.6% African American, and 1.1% other. In total, 63.4% lived in rural areas, 22.9% suburban, and 13.8% urban. There was no significant difference in OS or PFS by race (*p* = 0.15, 0.21) or place of residence (*p* = 0.39, 0.17). In addition, no difference in nonrelapse mortality, acute and chronic graft-versus-host disease (GVHD), and GVHD-free relapse-free survival (GRFS) was seen among the race or place of residence.

**Conclusion:**

Our study suggests that when appropriate access to HCT is given, there is no difference in outcomes based on race, ethnicity or place of primary residence. Further research is needed to further evaluate barriers for these patients to undergo transplant and help mitigate these barriers.

## Introduction

Allogeneic hematopoietic stem cell transplant (allo-HCT) plays a key role in the treatment of a number of both malignant and nonmalignant hematologic disorders and, in many cases, represents the sole curative option for patients. However, it is a complex procedure and is both costly and requires highly specialized, technologically advanced care that is only available in select healthcare centers. Due to cost and limited availability, minority populations are at risk for healthcare disparities in access to and outcomes of allo-HCT. Many factors contribute to a patient’s eligibility for transplant including appropriate disease status, adequate performance status and organ function, and having an available donor. Socioeconomic factors may also impair access to allo-HCT such as insurance coverage, social support, level of education, and income (1).

Impact of health disparities, including race and area of residence at the time of transplant, on the outcomes of allo-HCT has previously been studied with variable results. A study in 2014 by Khera et al. (2) compared outcomes in 296 patients undergoing allo-HCT between non-Hispanic Whites and minority patients, showing no significant difference in outcomes between the groups. Several CIMBTR studies prior to these suggested inferior outcomes for minority, particularly Black and Hispanic, patients undergoing allo-HCT (3, 4). Several studies have also evaluated the impact of primary location of residence on transplant outcomes. A CIMBTR study in 2010 (5) did not show any significant difference in outcomes based on urban and rural patients but did show significant outcomes based on income, while another study in 2016 by Khera et al. (6) did not show any significant difference in outcomes based on primary location of residence or distance from transplant center.

Due to these variable results in prior studies, we sought to evaluate the impact of race and primary location of residence on outcomes of allo-HCT, with the hypothesis that if given access to transplantation, outcomes are similar among these groups.

## Methods

### Study Design and Data Collection

A retrospective review was conducted of all adult patients (age ≥18 years old) who underwent allo-HCT at the Ohio State University from January 1, 1984 to December 31, 2018. The Institutional Review Board at the Ohio State University approved the study. All data were verified through medical chart review. Patients who were undergoing allo-HCT were included; for patients with multiple allo-HCTs, only their first allo-HCT was included in the analysis. Demographics were collected as well as transplant data collected including recipient and donor characteristics and disease characteristics. Due to the retrospective nature of the study, there were some missing data, particularly for those patients that were transplanted in 1980s and 1990s.

### Race and Geographic Location of Residence

Patients were divided into race for analysis utilizing self-reported race. Patients were classified as White, Black, and other. Due to the small percentage of patients that fell into the other category (1.1%), they were not included in the analyses by race. They were also stratified based on geographic location of residence into rural, suburban, and urban groups using zip code with 2018 population estimates used. Rural was defined as <1,000 people per square mile, suburban 1,000–3,000 people per square mile, and urban >3,000 people per square mile.

### Endpoints

The principal aim of this study was to evaluate differences in outcomes between patients based on race as well as primary geographic location of residence. Primary endpoints included progression free survival (PFS) and overall survival (OS). Patients who were living at assessment were censored at last follow-up. Secondary endpoints included nonrelapse mortality (NRM), acute and chronic graft-versus-host disease (aGVHD and cGVHD), GVHD-free relapse-free survival (GRFS), and transplant complications including infections, hemorrhagic cystitis, veno-occlusive disease (VOD), and pulmonary complications. Relapse was defined as recurrence of disease at any site. Grading of aGVHD was based on the Glucksberg grade (7). Standard definitions of cGVHD were used for diagnosis (8). GRFS was defined as the absence of grades III–IV aGVHD, cGVHD requiring systemic treatment, relapse, and death.

### Statistical Analysis

Patient, disease, and transplant-related characteristics were compared between the two groups using the Mann–Whitney *U* test for continuous variables, and Chi-squared or Fisher’s exact test for categorical variables. Probabilities of OS, PFS, and GRFS were calculated using the Kaplan–Meier (KM) method and compared using log-rank test. Cumulative incidence rates for NRM, aGVHD, and cGVHD were estimated and compared using Gray’s test accounting for competing risks. The competing risks for aGVHD and cGVHD were relapse or death, while the competing risk for NRM was death due to disease. Stata 14 was used for all the analyses, and statistical tests were two sided with significance level set at 0.05. Statistical tests were two sided with a statistical significance defined as *p* < 0.05. Kaplan–Meier curves were used to calculate PFS, OS, NRM, and GRFS.

## Results

### Patient, Disease, and Transplant Characteristics

A total of 1,943 patients were included in the study, with an overview of patient characteristics by race and geographic location shown in **Tables 1**, **2**, respectively. Median age at the time of transplant was 50 years old (range, 18–76) with the majority (59.6%) being men. The most common underlying diagnosis was acute myeloid leukemia (AML)/myelodysplastic syndrome (MDS), which made up 46.3% of the cohort with other common diagnoses including non-Hodgkin’s lymphoma (14.2%), acute lymphoblastic leukemia (11.8%), and chronic myeloid leukemia (10.1%). The majority of patients (94.2%) self-identified as White with 4.6% identifying as Black and 1.2% as other. The majority of patients also lived in rural areas at the time of transplant with 63.4% rural, 22.9% suburban, and 13.8% urban.

**Table 1:** Patient characteristics by race.

	White (*n* = 1,830)		Black (*n* = 89)		*p*-value
Age at HCT (median, range)	50.0	(18–76)	47.0	(22–71)	
Gender, patients	*N*	%	*N*	%	0.01
Male (M)	1,102	60.2	42	47.2	
Zip code					<0.001
Rural	1,176	64.3	43	48.3	
Suburban	414	22.6	23	25.8	
Urban	240	13.1	23	25.8	
Diagnosis					0.90
Aplastic anemia (AA)	35	1.9	1	1.1	
Acute lymphocytic leukemia (ALL)	213	11.6	12	13.5	
Acute myeloid leukemia (AML)	660	36.1	33	37.1	
Multiple myeloma (MM)	55	3.0	0	0.0	
Chronic myelogenous leukemia (CML)	185	10.1	11	12.4	
Chronic lymphocytic leukemia (CLL)	79	4.3	4	4.5	
Hodgkin’s disease (HD)	67	3.7	2	2.2	
Non-Hodgkin’s lymphoma (NHL)	260	14.2	14	15.7	
Myelodysplastic syndrome (MDS)	183	10.0	9	10.1	
Myeloproliferative disorder (MPD)	86	4.7	3	3.4	
Others	7	0.4	0	0.0	
KPS					0.25
<90	511	31.1	22	25.3	
≥90	1,131	68.9	65	74.7	
Donor type					<0.001
Matched related	861	47.0	36	40.4	
Matched unrelated	738	40.3	13	14.6	
Mismatch related	88	4.8	20	22.5	
Mismatch unrelated	143	7.8	20	22.5	
Haploidentical	57	3.1	19	21.3	
Cord blood	70	3.8	13	14.6	
Conditioning					0.52
MA	1,009	55.1	46	51.7	
RIC	821	44.9	43	48.3	
Comorbidity index					0.18
0–1	393	33.8	23	36.5	
2–3	433	37.2	17	27.0	
4–5	258	22.2	15	23.8	
5+	79	6.8	8	12.7	
Remission status at transplant					0.84
Blast and accelerated phase (AP)	60	3.7	2	2.6	
Chronic phase	117	7.3	8	10.3	
Complete response (CR)	787	49.0	40	51.3	
Stable disease (SD)	12	0.7	1	1.3	
Relapsed and refractory (R/R)	310	19.3	13	16.7	
Partial response (PR)	266	16.6	13	16.7	
N/A	53	3.3	1	1.3	
GVHD prophylaxis					0.95
Cyclosporine combination	226	15.9	13	17.6	
Tacrolimus combination	1,167	82.2	60	81.1	
Sirolimus	4	0.3	0	0.0	
Others	23	1.6	1	1.4	
CMV status recipient-donor					<0.001
Pos-Pos	380	24.5	40	48.2	
Pos-Neg	419	27.0	13	15.7	
Neg-Pos	240	15.5	12	14.5	
Neg-Neg	514	33.1	18	21.7	

HCT, hematopoietic stem cell transplant; SD, standard deviation; F, female; MA, myeloablative; RIC, reduced-intensity conditioning; KPS, Karnofsky performance status.

**Table 2:** Patient characteristics by location.

	Rural (*n* = 1,231)		Suburban (*n* = 444)		Urban (*n* = 268)		*p*-value
Age at HCT (median, range)	50.0	18–76	51.0	18–74	49.0	19–74	
Gender, patients	*N*	%	*N*	%	*N*	%	0.20
Male (M)	752	61.1	255	57.4	151	56.3	
Race, patients							0.003
White	1,176	95.7	414	93.5	240	89.6	
Black	43	3.5	23	5.2	23	8.6	
Others	10	0.8	6	1.4	5	1.9	
Diagnosis							0.59
Aplastic anemia (AA)	23	1.9	7	1.6	7	2.6	
Acute lymphocytic leukemia (ALL)	150	12.2	59	13.3	20	7.5	
Acute myeloid leukemia (AML)	454	36.9	155	34.9	96	35.8	
Multiple myeloma (MM)	34	2.8	14	3.2	7	2.6	
Chronic myelogenous leukemia (CML)	122	9.9	50	11.3	24	9.0	
Chronic lymphocytic leukemia (CLL)	45	3.7	23	5.2	17	6.3	
Hodgkin’s disease (HD)	44	3.6	17	3.8	9	3.4	
Non-Hodgkin’s lymphoma (NHL)	170	13.8	63	14.2	42	15.7	
Myelodysplastic syndrome (MDS)	130	10.6	34	7.7	31	11.6	
Myeloproliferative disorder (MPD)	55	4.5	21	4.7	13	4.9	
Others	4	0.3	1	0.2	2	0.7	
KPS							0.59
<90	337	30.4	130	32.8	73	29.4	
≥90	772	69.6	266	67.2	175	70.6	
Donor type							0.45
Matched related	590	47.9	188	42.3	130	48.5	
Matched unrelated	467	37.9	191	43.0	98	36.6	
Mismatch related	69	5.6	29	6.5	15	5.6	
Mismatch unrelated	105	8.5	36	8.1	25	9.3	
Haploidentical	50	4.1	20	4.4	11	4.1	
Cord blood	55	4.5	22	4.9	9	3.3	
Conditioning							0.40
MA	679	55.2	234	52.7	155	57.8	
RIC	552	44.8	210	47.3	113	42.2	
Comorbidity index							0.66
0–1	265	34.0	105	35.4	52	30.2	
2–3	291	37.4	99	33.3	71	41.3	
4–5	172	22.1	71	23.9	35	20.3	
5+	51	6.5	22	7.4	14	8.1	
Remission status at transplant							0.06
Blast or AP	34	3.1	21	5.4	7	3.1	
Chronic phase	81	7.5	27	6.9	17	7.4	
CR	536	49.6	201	51.0	108	46.8	
SD	4	0.4	4	1.0	5	2.2	
R/R	215	19.9	62	15.7	49	21.2	
PR	169	15.6	73	18.5	38	16.5	
N/A	42	3.9	6	1.5	7	3.0	
GVHD prophylaxis							0.65
Cyclosporine combination	155	16.2	51	14.4	34	16.6	
Tacrolimus combination	781	81.6	301	84.8	167	81.5	
Sirolimus	3	0.3	0	0.0	1	0.5	
Others	18	1.9	3	0.8	3	1.5	
CMV status recipient-donor							0.92
Pos-Pos	282	27.1	95	24.8	58	24.7	
Pos-Neg	270	25.9	103	26.9	67	28.5	
Neg-Pos	158	15.2	56	14.6	38	16.2	
Neg-Neg	332	31.9	129	33.7	72	30.6	

HCT, hematopoietic stem cell transplant; SD, standard deviation; F, female; MA, myeloablative; RIC, reduced-intensity conditioning; KPS, Karnofsky performance status.

White patients were more likely to live in rural areas than Black patients (64.3% vs. 48.3%; **Table 1**). A larger percentage of Black patients lived in urban areas (25.8% vs. 13.1%). White patients were more likely to be male with men representing 60.2% of the White cohort as compared with 47.2% of the Black cohort. White patients were more likely to undergo matched related or matched unrelated transplants with 47% of patients undergoing matched unrelated transplants and 40.3% matched related transplants as compared with 40.4% and 14.6%, respectively, in Black patients. There also was a significant difference in CMV seropositivity by race with higher rates of CMV positivity seen in Black patients in both recipients and donors (*p* = 0.03; *p* < 0.001). There was no significant difference between age at time of transplant, primary diagnosis, remission status at transplant, KPS, comorbidity index, or GVHD prophylaxis among the groups.

### Outcomes Based on Race

A summary of transplant outcomes based on race is shown in **Table 3**. There was no significant difference between median absolute neutrophil count (ANC) engraftment between White and Black patients. There was a significant difference in platelet engraftment with median platelet engraftment 27 days for Black and 19 days for White patients (*p* = 0.003). There was no significant different by race for posttransplant response with the majority of patients achieving a complete response (CR) in both groups. Rates of transplant-related complications were also similar between the two groups with comparable rates of pulmonary infections, VOD, bacteremia, viremia fungemia, and hemorrhagic cystitis.

**Table 3:** Transplant outcomes by race.

	White (*n* = 1,830)		Black (*n* = 89)		*p*-value
	Median	Range	Median	Range	
ANC engraftment	16	2–120	16	10–45	0.30
Platelet engraftment	19	8–758	27	10–130	0.003
	***N***	**%**	***N***	**%**	
Posttransplant response					0.85
CR	1,392	76.1	71	79.8	
Less than CR	158	8.6	6	6.7	
Progression	130	7.1	5	5.6	
Not available	150	8.2	7	7.9	
Pulmonary infection					0.84
No	1,504	83.5	75	84.3	
Yes	298	16.5	14	15.7	
VOD					0.78
No	1,566	86.6	78	87.6	
Yes	242	13.4	11	12.4	
Bacteremia in first D+100					0.81
No	1,057	61.1	49	59.8	
Yes	673	38.9	33	40.2	
Viremia in first D+100					0.23
No	965	55.5	40	48.8	
Yes	775	44.5	42	51.2	
Fungemia in first D+100					0.36
No	1,565	92.2	76	95.0	
Yes	132	7.8	4	5.0	
Hemorrhagic cystitis					0.45
No	1,605	90.0	77	87.5	
Yes	179	10.0	11	12.5	

ANC, absolute neutrophil count; CR, complete response; VOD, veno-occlusive disease; D+, day+.

**Figure 1:**
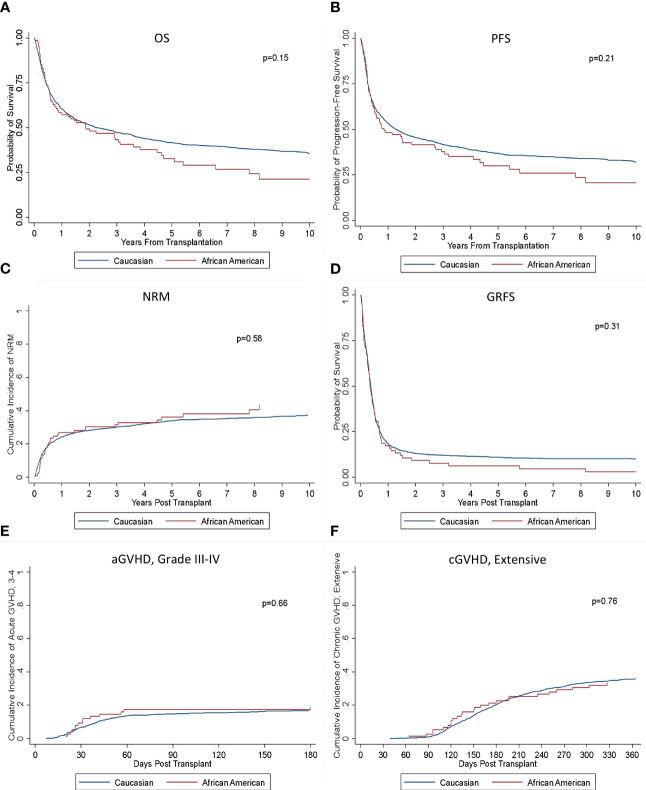
Outcomes based on race. There was no significant difference in **(A)** OS, **(B)** PFS, **(C)** NRM, **(D)** GRFS, **(E)** aGVHD III-IV, or **(F)** cGVHD (extensive) between the groups.

Among those alive, the median follow-up was 6.4 years. There was no significant difference in OS or PFS by race (*p* = 0.15; 0.21) (**Figure 1**). Median OS for Black patients was 1.9 years (95% confidence interval (CI): 0.8–3.6) compared with 2.3 years (95% CI: 1.9–2.9) for White patients with 3-, 5-, and 10-year OS of 43% versus 47%, 33% versus 42%, and 21% versus 36% for Black and White patients, respectively. Median PFS was 0.9 (95% CI: 0.5–2.7) and 1.3 years (95% CI: 1.1–1.6) with 3-, 5-, and 10-year PFS of 38% versus 42%, 30% versus 37%, and 21% versus 32% for Black and White patients, respectively. Cumulative incidence of relapse was also similar between the groups (*p* = 0.35). There was no significant difference in NRM between the groups (*p* = 0.58). Cumulative incidence of aGVHD was analyzed with no significant difference seen between rates of grades II–IV or III–IV aGVHD (*p* = 0.89; *p* = 0.66). There also was no significant difference seen between rates of cGVHD, both for extensive and limited and extensive alone (*p* = 0.96; *p* = 0.76). No significant difference in rates of GRFS was seen (*p* = 0.31).

### Outcomes Based on Geographic Location of Residence

An overview of transplant outcomes by geographic location of residence is provided in **Table 4**. There was no significant difference in time to ANC or platelet engraftment between the three groups (*p* = 0.98; 0.17). There also was no significant difference for posttransplant response among the groups with comparable rates of CR attained across the groups (*p* = 0.90). Rates of transplant-related complications were also similar with no significant difference seen between rates of pulmonary infection, VOD, bacteremia, viremia, fungemia, or hemorrhagic cystitis.

**Table 4:** Transplant outcomes by location.

	Rural (*n* = 1,231)		Suburban (*n* = 444)		Urban (*n* = 268)		*p*-value
	Median	Range	Median	Range	Median	Range	
ANC engraftment	16	2–120	16	2–32	16	6–51	0.98
Platelet engraftment	19	8–758	19	9–94	20	8–294	0.17
	***N***	**%**	***N***	**%**	***N***	**%**	
Posttransplant response							0.90
CR	941	76.4	336	75.7	209	78.0	
Less than CR	106	8.6	38	8.6	20	7.5	
Progression	86	7.0	29	6.5	21	7.8	
Not available	98	8.0	41	9.2	18	6.7	
Pulmonary infection							0.89
No	1,012	83.6	371	84.3	219	83.0	
Yes	199	16.4	69	15.7	45	17.0	
VOD							0.72
No	1,051	86.4	386	87.9	230	86.5	
Yes	165	13.6	53	12.1	36	13.5	
Bacteremia in first D+100							0.92
No	706	61.1	261	61.7	154	60.2	
Yes	449	38.9	162	38.3	102	39.8	
Viremia in first D+100							0.73
No	635	54.9	240	56.2	138	53.1	
Yes	522	45.1	187	43.8	122	46.9	
Fungemia in first D+100							0.51
No	1,045	92.4	389	93.3	228	90.8	
Yes	86	7.6	28	6.7	23	9.2	
Hemorrhagic cystitis							0.69
No	1,079	90.1	387	89.0	239	90.9	
Yes	119	9.9	48	11.0	24	9.1	

ANC, absolute neutrophil count; CR, complete response; VOD, veno-occlusive disease; D+, day+.

**Figure 2:**
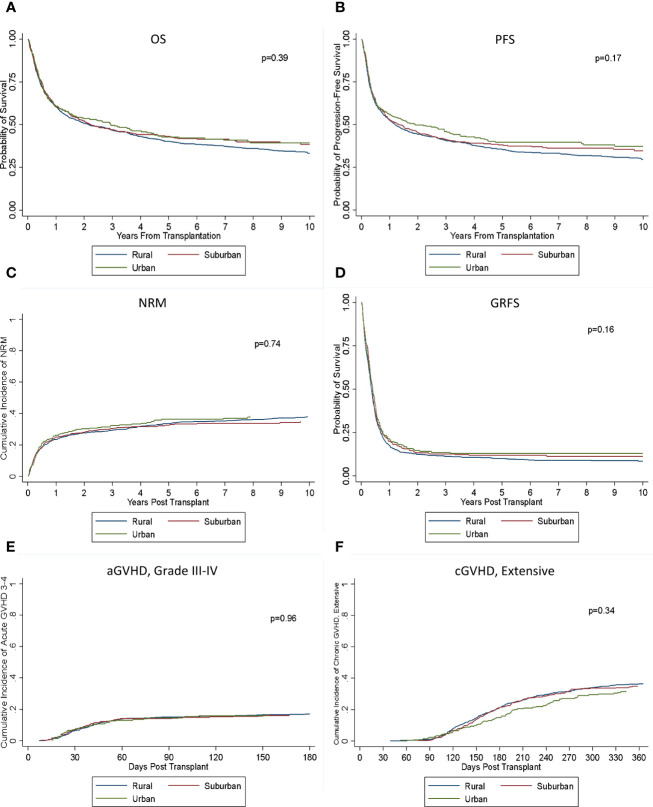
Outcomes based on location of residence. There was no significant difference in **(A)** OS, **(B)** PFS, **(C)** NRM, **(D)** GRFS, **(E)** aGVHD III–IV, or **(F)** cGVHD (extensive) between the groups.

There was no significant difference in OS or PFS between the rural, urban, and suburban groups (*p* = 0.39; *p* = 0.17) (**Figure 2**). Median OS in the three groups was 2.2 years (95% CI: 1.7–2.9), 2.9 years (95% CI: 1.6–4.5), and 2.2 years (95% CI: 1.6–3.6), and 3-, 5-, and 10-year OS was 50% versus 47% versus 47%, 40% versus 43% versus 43% and 33% versus 39% versus 39%, respectively. Median PFS were 2.2 years (95% CI: 1.7–2.9), 2.9 years (95% CI: 1.6–4.5), and 2.2 years (95% CI: 1.6–3.6) with 3-, 5-, and 10-year PFS of 46% versus 41% versus 41%, 36% versus 40% versus 38% and 30% versus 37% versus 35%, respectively. Cumulative incidence of relapse was also similar between the groups (*p* = 0.16), and there was no significant difference seen in NRM (*p* = 0.74). There was no significant difference in rates of grades II–IV aGVHD or grades III–IV aGVHD (*p* = 0.33; *p* = 0.96). Rates of both extensive and limited cGVHD and extensive cGVHD were also similar (*p* = 0.82; *p* = 0.34). There was no significant difference seen in GRFS (0.16).

## Discussion

Overall, our study did not show any significant differences in outcomes for patients undergoing allo-HCT based on race or primary area of residence. Prior studies have shown variable differences in outcomes between White and Black patients undergoing allo-HCT. One study by Hamilton et al. (9) evaluating 789 consecutive patients who underwent allo-HCT at Cleveland Clinic Taussig Cancer Institute did not show any significant difference in overall survival after performing a multivariate analysis. A prior study of patients undergoing HCT at Fred Hutchinson Cancer Research Center by Mielcarek et al. (10) showed a significant increase in mortality in Black patients undergoing allo-HCT as compared with White patients, which was thought to potentially be related to higher rates of severe aGVHD. Our study showed similar outcomes between White and Black patients including similar OS, PFS, NRM, and GRFS as well as similar rates of both acute and chronic GVHD and transplant complications.

Another recent landmark study by Bhatnagar et al. (11) evaluated survival of non-Hispanic Black and White adult patients with AML with mutational profiling obtained for 1,339 patients with AML that were treated on frontline Alliance for Clinical Trials in Oncology (Alliance) protocols. They found that Black patients had shorter survival compared with White patients, particularly in Black patients <60 years old. Fewer NPM1 and more IDH2 mutations were seen in these younger Black patients. Both socioeconomic factors and disease biology were thought to play a role in these disparate outcomes (11). Given that we did not see a difference in outcomes for our patients, this may suggest that Black patients with AML that do not undergo transplant have poorer outcomes overall than those that do. Further research is needed to further evaluate disease biology in Black and White patients and its relation to outcomes in AML patients, both in those who undergo transplant and those who do not.

While there were no significant differences in outcomes between White and Black patients, our study did demonstrate some key differences by race in those patients undergoing allo-HCT. Consistent with prior studies, a significantly higher proportion of Black patients underwent mismatched transplants. This likely reflects a lack of HLA-matched donors for Black patients, which has been demonstrated in prior studies (12–14). Based on data from the National Marrow Donor Program (NMDP), the chance of finding an HLA-A, HLA-B, HLA-C, and HLA-DRB1 high-resolution matched unrelated donor is only 19% for Black patients as compared with 75% for White patients of European descent (15). This significant disparity has been attributed to several factors including smaller donor pool, greater degree of HLA polymorphisms including many HLA haplotypes that are specific to Black patients, and higher attrition rates of non-White donors to the NMDP registry. Prior studies have suggested that barriers to blood and bone marrow donation among ethnic minorities may include ambivalence about donation, doubts and worries, and lack of education (13, 14). Strategies to improve recruitment and education in these groups will be important in the future to help ameliorate these barriers to donation to ultimately help increase their access to allo-HCT.

Black patients were also more likely to have a positive CMV serostatus with 63.9% of Black patients having a positive CMV serostatus compared with 51.4% of White patients. They also were more likely to have a donor that was seropositive for CMV. It has been well documented that race is associated with CMV seroprevalence, with Black adult patients shown to have twice the prevalence of CMV seropositivity as compared with White patients (16, 17). CMV infection is a major complication after allo-HCT and is associated with serostatus of the patient (18). However, CMV prophylaxis and close monitory for CMV reactivation posttransplant are common at our institution so this may account for the fact that increased CMV seropositivity in Black patients did not significantly impact overall outcomes. Interestingly, although prior studies have suggested significantly increased severity of aGVHD in Black patients undergoing allo-HCT, the rates of aGVHD that we saw were similar among the groups with no significant difference seen. This may be due to the small population of Black patients included in our study but may also reflect the fact that although genetic diversity has previously been shown to play a role, having access to care eliminated those differences.

Our study also did not show any significant difference in outcomes based on primary place of residence, which is consistent with prior studies. There also were no significant differences in hospitalizations, infections, complications, or rates of acute or chronic GVHD. One prior CIMBTR study by Loberiza et al. (5) compared outcomes of rural and urban patients with leukemia or MDS undergoing unrelated donor HCT. The study included 6,140 patients undergoing allo-HCT from 1995 to 2004 with 19% of patients from rural areas and 81% from urban areas. No significant difference was seen in outcomes between the two groups; however, income was found to be independently associated with outcomes. This study did have a significantly lower percentage of rural patients as compared with our study (19% vs. 63.4% in our study) and also only included unrelated donor transplants undergoing MA regimens. Another smaller study by Rao et al. (19) evaluated differences in outcomes between urban and rural patients undergoing both autologous (*n* = 1,739) and HLA-identical sibling allo-HSCT (*n* = 267) from 1983 to 2004. They found a higher relative risk of death in patients from rural areas undergoing autologous HCT but did not find a significant difference in outcomes for those patients undergoing allo-HCT.

Our study is limited by the retrospective nature of study design. We were unable to assess the rates of referrals for transplant and whether these are similar between patients based on race and primary place of residence. The patient population was also limited in diversity of race with only a small percentage of Black and other patients included in the study, with Black patients only representing 4.6% of the total population. The lack of significant diversity in our patient population is not entirely surprising given that our center primarily draws from Ohio, West Virginia, Indiana, and Kentucky, a population which is predominately non-Hispanic White based on Census Data (20). However, the percentage of ethnic minorities is still lower than would be expected which may reflect a lower referral rate for these patients. The number of Black patients was relatively consistent with prior larger CIBMTR studies (3, 21); however, we recognize that the population of Black patients in the study was small and thus the study may not be adequately powered to detect a difference in outcomes. Due to the retrospective nature of the study, certain socioeconomic factors were also unable to be assessed such as income, level of education, insurance, compliance, and genetic polymorphisms, which may have a significant impact on outcomes. While our study did have some key limitations, it is a large analysis.

Overall, our study suggests that while underserved populations, including those living in rural areas and Black patients, may initially have less access to allo-HCT, when they do undergo allo-HCT outcomes are similar to their counterparts. This is important because prior research has shown that providers are less likely to refer certain groups of patients for allo-HCT, including ethnic minorities, than their counterparts (22). Based on the results of our study, we would encourage providers to refer these patients for allo-HCT evaluation as aggressively as nonminority patients. Further research should evaluate barriers to transplant referral and further assess other factors that may affect rates of transplants in these groups. Recruitment of HLA-matched Black donors as well as advancement of haploidentical transplants for minority patients may also represent viable options to help increase accessibility of allo-HCT to these underserved populations, as haploidentical transplants have been shown to have superior survival outcomes in Black as compared with White patients (23). A multi-institutional study would be beneficial in the future to optimize sample size to further evaluate the impact of race on outcomes in allo-HCT.

## Data Availability Statement

The raw data supporting the conclusions of this article will be made available by the authors, without undue reservation.

## Ethics Statement

The studies involving human participants were reviewed and approved by The Institutional Review Board at The Ohio State University. Written informed consent for participation was not required for this study in accordance with the national legislation and the institutional requirements.

## Author Contributions

Conception and design: YE, NS, and AS. Collection and assembly of data: AS and JJ. Data analysis and interpretation: QZ, AS, NS, and YE. Manuscript writing: AS, NS, and YE. Scientific input and critical comments: all authors. All authors listed have made a substantial, direct, and intellectual contribution to the work and approved it for publication.

## Conflict of Interest

The authors declare that the research was conducted in the absence of any commercial or financial relationships that could be construed as a potential conflict of interest.

## Publisher’s Note

All claims expressed in this article are solely those of the authors and do not necessarily represent those of their affiliated organizations, or those of the publisher, the editors and the reviewers. Any product that may be evaluated in this article, or claim that may be made by its manufacturer, is not guaranteed or endorsed by the publisher.

## References

[1] Majhail NS Omondi NA Denzen E Murphy EA Rizzo JD. Access to Hematopoietic Cell Transplantation in the United States. Biol Blood Marrow Transplant (2010) 16(8):1070–5. doi: 10.1016/j.bbmt.2009.12.529 PMC3144674 20036337

[2] Khera N Chang YH Slack J Fauble V Leis JF Noel P. Impact of Race and Ethnicity on Outcomes and Health Care Utilization After Allogeneic Hematopoietic Cell Transplantation. Leuk Lymphoma (2015) 56(4):987–92. doi: 10.3109/10428194.2014.941834 25012944

[3] Baker KS Davies SM Majhail NS Hassebroek A Klein JP Ballen KK. Race and Socioeconomic Status Influence Outcomes of Unrelated Donor Hematopoietic Cell Transplantation. Biol Blood Marrow Transplant (2009) 15(12):1543–54. doi: 10.1016/j.bbmt.2009.07.023 PMC2775819 19896078

[4] Ballen KK Klein JP Pedersen TL Bhatla D Duerst R Kurtzberg J. Relationship of Race/Ethnicity and Survival After Single Umbilical Cord Blood Transplantation for Adults and Children With Leukemia and Myelodysplastic Syndromes. Biol Blood Marrow Transplant (2012) 18(6):903–12. doi: 10.1016/j.bbmt.2011.10.040 PMC3874400 22062801

[5] Loberiza FR Jr. Lee SJ Klein JP Hassebroek A Dehn JG Frangoul HA. Outcomes of Hematologic Malignancies After Unrelated Donor Hematopoietic Cell Transplantation According to Place of Residence. Biol Blood Marrow Transplant (2010) 16(3):368–75. doi: 10.1016/j.bbmt.2009.10.028 PMC2822013 19879951

[6] Khera N Gooley T Flowers MED Sandmaier BM Loberiza F Lee SJ. Association of Distance From Transplantation Center and Place of Residence on Outcomes After Allogeneic Hematopoietic Cell Transplantation. Biol Blood Marrow Transplant (2016) 22(7):1319–23. doi: 10.1016/j.bbmt.2016.03.019 PMC4905774 27013013

[7] Glucksberg H Storb R Fefer A Buckner CD Neiman PE Clift RA. Clinical Manifestations of Graft-Versus-Host Disease in Human Recipients of Marrow From HL-A-Matched Sibling Donors. Transplantation (1974) 18(4):295–304. doi: 10.1097/00007890-197410000-00001 4153799

[8] Filipovich AH Weisdorf D Pavletic S Socie G Wingard JR Lee SJ. National Institutes of Health Consensus Development Project on Criteria for Clinical Trials in Chronic Graft-Versus-Host Disease: I. Diagnosis and Staging Working Group Report. Biol Blood Marrow Transplant (2005) 11(12):945–56. doi: 10.1016/j.bbmt.2005.09.004 16338616

[9] Hamilton BK Rybicki L Sekeres M Kalaycio M Hanna R Sobecks R. Racial Differences in Allogeneic Hematopoietic Cell Transplantation Outcomes Among African Americans and Whites. Bone Marrow Transplant (2015) 50(6):834–9. doi: 10.1038/bmt.2015.44 25798671

[10] Mielcarek M Gooley T Martin PJ Chauncey TR Young BA Storb R. Effects of Race on Survival After Stem Cell Transplantation. Biol Blood Marrow Transplant (2005) 11(3):231–9. doi: 10.1016/j.bbmt.2004.12.327 15744242

[11] Bhatnagar B Kohlschmidt J Mrozek K Zhao Q Fisher JL Nicolet D. Poor Survival and Differential Impact of Genetic Features of Black Patients With Acute Myeloid Leukemia. Cancer Discovery (2021) 11(3):626–37. doi: 10.1158/2159-8290.CD-20-1579 PMC7933110 33277314

[12] Dehn J Arora M Spellman S Setterholm M Horowitz M Confer D. Unrelated Donor Hematopoietic Cell Transplantation: Factors Associated With a Better HLA Match. Biol Blood Marrow Transplant (2008) 14(12):1334–40. doi: 10.1016/j.bbmt.2008.09.009 PMC3319684 19041054

[13] Laver JH Hulsey TC Jones JP Gautreaux M Barredo JC Abboud MR. Assessment of Barriers to Bone Marrow Donation by Unrelated African-American Potential Donors. Biol Blood Marrow Transplant (2001) 7(1):45–8. doi: 10.1053/bbmt.2001.v7.pm11215698 11215698

[14] Switzer GE Bruce JG Myaskovsky L DiMartini A Shellmer D Confer DL. Race and Ethnicity in Decisions About Unrelated Hematopoietic Stem Cell Donation. Blood (2013) 121(8):1469–76. doi: 10.1182/blood-2012-06-437343 PMC3578960 23258921

[15] Gragert L Eapen M Williams E Freeman J Spellman S Baitty R. HLA Match Likelihoods for Hematopoietic Stem-Cell Grafts in the U.S. Registry. N Engl J Med (2014) 371(4):339–48. doi: 10.1056/NEJMsa1311707 PMC5965695 25054717

[16] Cannon MJ Schmid DS Hyde TB. Review of Cytomegalovirus Seroprevalence and Demographic Characteristics Associated With Infection. Rev Med Virol (2010) 20(4):202–13. doi: 10.1002/rmv.655 20564615

[17] Colugnati FA Staras SA Dollard SC Cannon MJ. Incidence of Cytomegalovirus Infection Among the General Population and Pregnant Women in the United States. BMC Infect Dis (2007) 7:71. doi: 10.1186/1471-2334-7-71 17605813 PMC1925089

[18] Boeckh M Nichols WG. The Impact of Cytomegalovirus Serostatus of Donor and Recipient Before Hematopoietic Stem Cell Transplantation in the Era of Antiviral Prophylaxis and Preemptive Therapy. Blood (2004) 103(6):2003–8. doi: 10.1182/blood-2003-10-3616 14644993

[19] Rao K Darrington DL Schumacher JJ Devetten M Vose JM Loberiza FR Jr. Disparity in Survival Outcome After Hematopoietic Stem Cell Transplantation for Hematologic Malignancies According to Area of Primary Residence. Biol Blood Marrow Transplant (2007) 13(12):1508–14. doi: 10.1016/j.bbmt.2007.09.006 18022581

[20] Bureau USC. Ohio Population Estimates by Age, Sex, Race, and Hispanic Origin. (2018).

[21] Serna DS Lee SJ Zhang MJ Baker k S Eapen M Horowitz MM. Trends in Survival Rates After Allogeneic Hematopoietic Stem-Cell Transplantation for Acute and Chronic Leukemia by Ethnicity in the United States and Canada. J Clin Oncol (2003) 21(20):3754–60. doi: 10.1200/JCO.2003.03.133 14551294

[22] Pidala J Craig BM Lee SJ Majhail N Quinn G Anasetti C. Practice Variation in Physician Referral for Allogeneic Hematopoietic Cell Transplantation. Bone Marrow Transplant (2013) 48(1):63–7. doi: 10.1038/bmt.2012.95 PMC3549547 22705801

[23] Solomon SR Zhang X Holland HK Morris LE Solh M Bashey A. Superior Survival of Black Versus White Patients Following Post-Transplant Cyclophosphamide-Based Haploidentical Transplantation for Adults With Hematologic Malignancy. Biol Blood Marrow Transplant (2018) 24(6):1237–42. doi: 10.1016/j.bbmt.2018.01.024 29378303

